# Community coalition efforts to prevent childhood obesity: two-year results of the Shape Up Under 5 study

**DOI:** 10.1186/s12889-023-15288-5

**Published:** 2023-03-20

**Authors:** Christina D. Economos, Larissa Calancie, Ariella R. Korn, Steven Allender, Julia M. Appel, Peter Bakun, Erin Hennessy, Peter S. Hovmand, Matt Kasman, Melanie Nichols, Mark C. Pachucki, Boyd A. Swinburn, Alison Tovar, Ross A. Hammond

**Affiliations:** 1 Friedman School of Nutrition Science and Policy, Tufts University, Tufts University, 150 Harrison Ave., Boston, MA 02111 USA; 2 Institute for Health Transformation, Deakin University, Geelong, Australia; 3 Center for Community Health Integration, Case Western Reserve University, Cleveland, OH USA; 4 Economic Studies, Brookings, Washington, D.C. USA; 5 Sociology and Computational Social Science Institute, University of Massachusetts, Amherst, MA USA; 6 School of Population Health, University of Auckland, Auckland, New Zealand; 7 Behavioral and Social Sciences, Brown University, Providence, RI USA; 8 Brown School, Washington University in St Louis, St Louis, MO USA; 9 Santa Fe Institute, Santa Fe, NM USA

**Keywords:** Childhood obesity, Coalition, Communication campaign, Community health, Public health, Nutrition, Social network, Intervention

## Abstract

**Background:**

Cross-sector collaborations and coalitions are promising approaches for childhood obesity prevention, yet there is little empirical evidence about *how* they affect change. We hypothesized that changes in knowledge of, and engagement with, childhood obesity prevention among coalition members can diffuse through social networks to influence policies, systems, and environments.

**Methods:**

We studied a community coalition (*N* = 16, Shape Up Under 5 “SUU5 Committee”) focused on early childhood obesity prevention in Somerville, MA from 2015–17. Knowledge, engagement, and social network data were collected from Committee members and their network contacts (*n* = 193) at five timepoints over two years. Policy, systems, and environment data were collected from the SUU5 Committee. Data were collected via the validated COMPACT Stakeholder-driven Community Diffusion survey and analyzed using regression models and social network analysis.

**Results:**

Over 2 years, knowledge of (*p* = 0.0002), and engagement with (*p* = 0.03), childhood obesity prevention increased significantly among the SUU5 Committee. Knowledge increased among the Committee’s social network (*p* = 0.001). Significant changes in policies, systems, and environments that support childhood obesity prevention were seen from baseline to 24 months (*p* = 0.003).

**Conclusion:**

SUU5 had positive effects on “upstream” drivers of early childhood obesity by increasing knowledge and engagement. These changes partially diffused through networks and may have changed “midstream” community policies, systems, and environments.

**Supplementary Information:**

The online version contains supplementary material available at 10.1186/s12889-023-15288-5.

## Background

Almost 14% of 2–5 years old children have obesity in the United States (US) [1]. Children with obesity in early childhood are likely to have obesity as adolescents and adults, increasing their risk for diabetes, hypertension, cardiovascular disease, and certain cancers [2, 3]. Moreover, obesity in this age group suggests the presence of unhealthy eating and physical activity behaviors that could be modified with early interventions [4]. Obesity rates are significantly higher among Hispanic and African American children compared to White children, highlighting health disparities that can develop early in life [5, 6]. Obesity in early childhood is influenced by many interacting factors, including families’ ability to access and afford healthy foods, caregivers’ child-feeding beliefs, access to safe places for active play, childcare center policies and practices, and children’s changing taste preferences [7, 8].

A number of evidence-based obesity prevention policies and interventions have been developed and are readily available for adoption [9, 10]. A systematic review of interventions aiming to promote healthy weights in children 2–5 years old found that interventions that included the following three components were more successful: encouraging parents to praise healthy behaviors in their children, educating parents about the importance of reducing screen time for their children, and engaging healthcare providers in intervention content delivery [11, 12]. However, parents, healthcare providers, and early childcare education professionals report barriers in adopting evidence-based obesity prevention strategies without environmental and policy changes within the broader system [13–15].

Engaging cross-sector collaborations, which include community coalitions and partnerships across multiple sectors (e.g., government, healthcare, community-based organizations) [16–18] are promising approaches for implementing childhood obesity prevention strategies and interventions in community settings [19, 20]. A review of interventions involving community coalitions found elements of Community-based Participatory Research were present in all 13 included studies, and five reported improvements in anthropometric, behavioral, or policy outcomes [20]. The review found successful studies demonstrated the importance of community engagement in community-based childhood obesity prevention research [20].

**Fig. 1:**
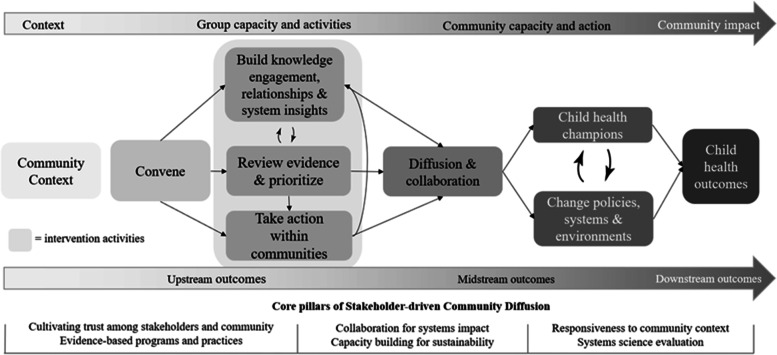
Stakeholder-driven Community Diffusion theory-informed intervention [21]

While engaging cross-sector collaborations and coalitions are promising approaches for addressing early childhood obesity in communities, there is little empirical evidence about *how* these collaborations work to effect change. Practical, evidence-based theories for how to work across sectors to change policies, systems, and environments (PSE) to reduce early childhood obesity are needed. We previously developed the Stakeholder-driven Community Diffusion (SDCD) theory (Fig. 1), which posits that a multi-sector group can diffuse knowledge and engagement across both existing and new relationship networks, thus facilitating changes in policies, systems, and environments that influence obesity [21–23]. SDCD was informed by Community-based Participatory Research [24], Community Coalition Action Theory [25], Diffusion of Innovations [26], and systems science [27]. In SDCD, *knowledge* and *engagement* are critical upstream mechanisms that provide a foundation and motivation for creating long-term, widespread, sustainable change within communities [21, 28]. SDCD was developed as part of the COMPACT study (R01HL115485) that integrated multiple systems science methods to study and support dynamics within community coalitions that fostered the adoption, implementation, and dissemination of evidence-based childhood obesity prevention strategies across multiple community sectors [28]. The goal of SDCD-informed interventions are to catalyze whole-of-community change with multi-level, multicomponent obesity prevention strategies that are implemented in a variety of settings across a community [29].

Previous research has provided suggestive evidence that similar interventions in other settings have had measurable impact, and has examined the SDCD causal mechanism with retrospective analyses [30]. The purpose of this study is to conduct a prospective analysis of an SDCD-driven intervention, testing the following three hypotheses: 1) knowledge of and engagement with early childhood obesity prevention efforts will increase among Committee members; 2) knowledge and engagement will increase among Committee members’ networks; and 3) policies, systems, and environments in the community where the Committee works will become more supportive of early childhood obesity prevention efforts over the 2-year study period.

## Methods

### Shape up under 5 study

Shape Up Under 5 (SUU5) was a two-year study (2015–2017) designed to test the SDCD theory as a systems approach to early childhood obesity prevention in Somerville, MA [21]. At the time of the study, the prevalence of obesity among children under five years old participating in the Massachusetts Pediatric Nutrition Surveillance System was 14.4%, which was slightly higher than the national obesity prevalence (13.4%) [31]. Central to SUU5 was the formation of a 16-person coalition (“SUU5 Committee”) composed of early childhood education and care, healthcare, parks and recreation, local public health department, and public school system representatives [21]. The Committee met 16 times in total – every 4 to 6 weeks – facilitated by a research team from Tufts University and advised by Community-based System Dynamics (CBSD) experts. CBSD is a method for engaging community members in developing a dynamic model of a complex problem and potential solutions; CBSD is rooted in CBPR and system dynamics [32]. CBSD projects often utilize group model building (GMB), which are structured activities that engage groups to build models of a dynamic problem in order to create a shared understanding of system structures that drive that problem over time and build consensus on how to address the problem [32, 33]. SUU5 employed CBSD and GMB activities because they are promising practices for building a shared vision of how to move forward to address complex social challenges while building systems thinking capabilities among participants [32, 34].

The intervention had three phases: 1) reviewing evidence-based strategies for addressing early childhood obesity; 2) GMB to develop a shared understanding of the systems driving early childhood obesity locally and to prioritize actions to address the problem grounded in community context; and 3) stakeholders taking action to address early childhood obesity in their community, with support from the research team [21].

A detailed description of GMB activities and their effects on participants, and the structure and change of the SUU5 social networks over time are presented elsewhere [35, 36]. Briefly, SUU5 Committee members decided to prioritize early childhood obesity prevention, using evidence-based policy and environmental change strategies, within their own organizations and [35] and the network’s evolving membership provided access to a wide range of resources, ideas, and an ability to broadly disseminate intervention messages [36].

### Data collection – knowledge and engagement

We collected data about perceived knowledge and engagement from SUU5 Committee members. Knowledge and engagement data using the COMPACT SDCD survey; details on the development and validation of the can be found elsewhere [36–38]. The survey was administered at baseline and then every six months online with a total of five measurements over the two-year study period [21]. Knowledge was defined as stakeholders’ perceived understanding of various factors related to early childhood obesity prevention efforts, and comprised of 18 items on a 5-point Likert scale across five domains: (i) the problem of early childhood obesity, (ii) modifiable intervention factors, (iii) their own role and the role of others, (iv) how to intervene sustainably, and (v) available resources [37]. Engagement was defined as a latent construct broadly representing stakeholders’ enthusiasm and agency for addressing early childhood obesity. The engagement scale included 25 items on the same 5-point response scale across the following five domains: (i) dialogue and mutual learning, (ii) flexibility, (iii) influence and power, (iv) leadership and stewardship, and (v) trust [37].

### Data collection – social networks and diffusion of knowledge and engagement

We also surveyed committee members’ early childhood obesity prevention social contacts, and collected the same responses regarding perceived knowledge and engagement to test the diffusion mechanism posited in the SDCD theory. At each SDCD survey timepoint the Committee identified up to 20 people with whom they discussed early childhood obesity prevention in the past three months [37]; nominated discussion partners are referred to as “first-degree alters” [39]. To measure knowledge and engagement, first-degree alters were invited to complete the COMPACT SDCD survey including network data at each round of data collection after they were nominated by a Committee member [36].

### Data collection – policies, systems, and environments

As proposed in the SDCD theory, knowledge and engagement spreads through social networks and promotes the implementation of PSE changes [21]. PSE change was assessed via an online survey of respondents’ perception of policy, systems, and environmental changes in Somerville at three times: baseline, 12 months, and 24 months [21]. The PSE survey was informed by existing surveys assessing the nutrition and physical activity environment in early childhood education settings and the research team’s knowledge of environments in Somerville [40–42]. PSE changes that were captured in the survey included: (i) Somerville’s commitment to different aspects of the built environment (e.g., workplace accommodations for breastfeeding); (ii) current use of public places or programs by Somerville residents (e.g., public parks and swimming pools); (iii) availability of family health materials at workplaces; and (iv) training and support for health promoting activities.

There were three survey modules. All Committee members were given module A (*N* = 16), which asked questions about the built environment and workplace prioritization of early childhood obesity prevention. Committee members who provided direct service to children and/or families (e.g., pediatrician) were given modules A and B (*n* = 12), which included questions about the type and frequency of distribution of resources for clients. Committee members who worked in a childcare setting (e.g., center-based childcare provider) were given modules A, B, and C, which included questions about food and menu policies in childcare settings (*n* = 4). We decided which Committee members would receive which modules (i.e., either A only; A and B; or A, B, and C) a priori, based on their occupation at the beginning of the study. The instructions in the survey also suggested that respondents review relevant materials (e.g., food menus, staff manuals, parent handbooks, or other policy documents or guidelines) before they completed the survey. Additional information can be found in Appel 2019, and request for further information about this survey can be directed to the corresponding author. Response options were presented as Likert scales where the scales aligned with the question format (e.g., “In general, how would you rate Somerville’s involvement in the following topics related to prenatal and early childhood health?”). The responses for that are on a 5-point Likert scale. We classified “I do not know” responses as missing.

### Data collection – community campaign reach

Through the regular Committee meetings using GMB and other structured activities, the SUU5 Committee determined there was a need for coordinated messaging about early childhood obesity prevention strategies that target multiple audiences (e.g., parents, caregivers, and health service providers) and that resonated with the diverse population in Somerville. To fill this need, the Committee and members of the research team at Tufts University created an evidence-informed community communications campaign called *Small Steps: Eat, Play, Sleep* (adapted from the 9–5-2–1-0 campaign to include recommendations for children under the age of five [43, 44]). The campaign included posters, three different brochures with age-appropriate recommendations and information for children in three age ranges (birth – nine months, nine months – three years, and three – five years) informational videos, activities for an annual Mayor’s Wellness Challenge and a tip-sheet for health and childcare providers. The poster and brochures were made available in four languages: English, Spanish, Portuguese, and Haitian Creole [21, 35], at least one of which is spoken by about 85% of Somerville residents. The informational videos were made available in English and Spanish. The Committee and research team worked together to ensure that the physical materials were displayed in multiple locations throughout the city, and that the digital resources were available on city websites [45]. To estimate the reach of the campaign, we reviewed logs that the Committee filled out describing where they placed the materials and an estimate of how many people might have seen them and we surveyed first-degree alters nominated by the SUU5 Committee. The reach survey asked participants if they saw campaign materials, what materials they saw, and where the materials were located.

### Data analysis – knowledge and engagement

For knowledge and engagement, we calculated domain-specific scores by summing responses within domains using a 5-point Likert scale from strongly disagree (1) to strongly agree (5), dividing by the number of items, and then scaled from 0 to 1 to enable direct comparisons between domains. We also calculated composite scores for knowledge and engagement by averaging domain scores. Scores were calculated for each of the five survey rounds for Committee members and first-degree alters. To examine knowledge and engagement changes over time, we used mixed effects regression models with random intercept and trend allowing individual subject effects to vary over time. Pairwise comparisons of mean knowledge and engagement scores by survey round were made using ANOVA with the Tukey method for type 1 error adjustment. All analyses were conducted with SAS 9.4.

### Data analysis – social networks and diffusion of knowledge and engagement

We constructed a “network neighborhood” variable for each Committee member’s cluster of first-degree alters by taking the average knowledge and engagement score of the cluster of alters named by the Committee member [46]. Network neighborhood scores for both knowledge and engagement were calculated at each survey round. All first-degree alters were carried forward to the next round, creating a cumulative network. For any first-degree alter missing a survey round, last observation carried forward was used as a conservative approach to impute missing knowledge and engagement scores. Network diagrams with knowledge and engagement scores were created using the R-igraph package implemented in RStudio (v1.2.5019) [46]. Analysis of potential diffusion patterns beyond the first-degree alters (e.g., further into the community networks) was conducted separately using simulation approaches and is reported in a related paper [47].

### Data analysis – policies, systems, and environments

PSE scores were calculated by summing the raw score for all completed sections and dividing it by the total possible score for the completed sections. Change in PSE scores was analyzed using the same methods as knowledge and engagement change. In addition, the relationship of PSE scores to knowledge and engagement score was analyzed using linear regression, regressing PSE score on time and knowledge or engagement score, adjusting for years of Committee member experience.

## Results

The Committee was highly educated (75% graduate degree), mostly white (94%) and Hispanic (6%), and predominately female (88%). The mean age was 50 years old (range: 29 to 70 years old), and years of experience in their occupations ranged from 4 to 44 years (mean = 20 years). First degree alters were demographically similar; 62% held graduate degrees, 89% were white, 10% were Hispanic, and 85% were female. The mean age was 43 (range: 21 to 74 years old) and occupational experience ranged from 0 to 37 years (mean = 13 years).

### Hypothesis 1: Knowledge and engagement change among committee members

Overall knowledge scores were higher at every follow-up (6, 12, 18 and 24 months), compared to baseline, although the difference was not significant after 6 months (Table 1). Increases in knowledge of available *resources* was significantly higher at every round compared to baseline. Knowledge of the *problem*, *intervention factors*, and *sustainability* were significantly higher than baseline at some, but not all, rounds. Overall engagement increased from baseline to 24 months (β = 0.011 (0.005), *p* = 0.03). Within engagement domains, *influence and power* increased (β = 0.029 (0.011), *p* = 0.01) and *flexibility* increased between baseline and 12 months (mean difference = 0.089, CI = 0.00087, 0.1776).

**Table 1:** Composite- and domain-level knowledge and engagement scores among Shape Up Under 5 Committee members (*n* = 16) and first-degree alters over the two-year pilot study period. Mean (SD) knowledge and engagement scores range from 0 (low) to 1 (high). The number of first-degree alters who were surveyed expanded at each measurement time as new alters were nominated by committee members

	Measurement time						
**Committee members (*n* = 16)**	Baseline	6 months	12 months	18 months	24 months	β (SE)	*p*-value
**Knowledge**	0.67 (0.07)	0.73 (0.09)	0.73 (0.08)*	0.78 (0.08)*	0.77 (0.09)*	**0.027 (0.006)**	**0.0002**
* Problem*	0.75 (0.11)	0.80 (0.10)	0.79 (0.12)*	0.82 (0.11)	0.83 (0.10)*	**0.021 (0.007)**	**0.007**
* Intervention factors*	0.73 (0.11)	0.78 (0.15)	0.80 (0.12	0.80 (0.10)	0.78 (0.14)	**0.014 (0.005)**	**0.020**
* Roles*	0.60 (0.14)	0.59 (0.19)	0.64 (0.12)	0.68 (0.15)	0.70 (0.13)	**0.029 (0.011)**	**0.010**
* Sustainability*	0.80 (0.13)	0.86 (0.11)*	0.84 (0.11)	0.87 (0.10)	0.85 (0.10)	0.010 (0.008)	0.200
* Resources*	0.46 (0.11)	0.58 (0.19)*	0.59 (0.16)*	0.72 (0.12)*	0.67 (0.17)*	**0.058 (0.011)**	**0.0002**
**Engagement**	0.73 (0.14)	0.75 (0.12)	0.80 (0.12)*	0.79 (0.11)*	0.76 (0.13)	**0.011 (0.005)**	**0.030**
* Dialogue & mutual learning*	0.82 (0.12)	0.84 (0.14)	0.85 (0.12)	0.88 (0.11)	0.82 (0.11)	0.006 (0.006)	0.300
* Flexibility*	0.76 (0.16)	0.77 (0.15)	0.84 (0.13)*	0.83 (0.12)	0.78 (0.16)	0.014 (0.009)	0.200
* Influence & power*	0.55 (0.30)	0.58 (0.24)	0.67 (0.23)	0.68 (0.21)	0.64 (0.29)	**0.029 (0.011)**	**0.010**
* Leadership & stewardship*	0.77 (0.13)	0.75 (0.18)	0.78 (0.12)	0.76 (0.10)	0.74 (0.10)	-0.002 (0.006)	0.600
* Trust*	0.78 (0.15)	0.81 (0.11)	0.86 (0.14)	0.82 (0.11)	0.82 (0.11)	0.009 (0.007)	0.200
**First degree alters**	*n* = 65	*n* = 133	*n* = 165	*n* = 186	*n* = 193		
**Knowledge**	0.70 (0.10)	0.72 (0.10)	0.72 (0.09)	0.73 (0.11)	0.74 (0.11)	**0.008 (0.002)**	**0.001**
* Problem*	0.72 (0.12)	0.77 (0.11)	0.78 (0.11)	0.79 (0.12)	0.80 (0.12)	**0.012 (0.003)**	**0.0001**
* Intervention factors*	0.76 (0.11)	0.79 (0.13)	0.78 (0.12)	0.78 (0.13)	0.78 (0.13)	-0.001 (0.003)	0.843
* Roles*	0.60 (0.14)	0.66 (0.16)	0.63 (0.17)	0.64 (0.17)	0.67 (0.18)	0.007 (0.004)	0.090
* Sustainability*	0.82 (0.11)	0.80 (0.13)	0.83 (0.13)	0.82 (0.12)	0.83 (0.14)	0.004 (0.003)	0.153
* Resources*	0.58 (0.15)	0.56 (0.15)	0.60 (0.16)	0.63 (0.20)	0.63 (0.22)	**0.018 (0.005)**	**0.0003**
**Engagement**	0.76 (0.07)	0.80 (0.09)	0.77 (0.10)	0.78 (0.10)	0.78 (0.10)	-0.001 (0.002)	0.662
* Dialogue & mutual learning*	0.84 (0.08)	0.88 (0.11)	0.86 (0.11)	0.87 (0.13)	0.87 (0.12)	0.001 (0.003)	0.747
* Flexibility*	0.73 (0.11)	0.83 (0.11)	0.79 (0.15)	0.80 (0.13)	0.80 (0.14)	0.003 (0.003)	0.245
* Influence & power*	0.62 (0.16)	0.65 (0.18)	0.60 (0.19)	0.61 (0.22)	0.60 (0.22)	**-0.013 (0.005)**	**0.004**
* Leadership & stewardship*	0.79 (0.09)	0.82 (0.11)	0.78 (0.11)	0.79 (0.11)	0.80 (0.11)	0.002 (0.002)	0.427
* Trust*	0.80 (0.10)	0.80 (0.11)	0.80 (0.12)	0.82 (0.13)	0.83 (0.14)	**0.009 (0.003)**	**0.004**

Betas estimated using a mixed effects regression model with random intercept and trend allowing individual subject effects to vary over time

Changes between baseline and a follow-up time point with significant *p*-values (*p* < 0.05) are bolded

^*^p < 0.05, change from baseline using ANOVA with the Tukey method for type 1 error

### Hypothesis 2: Knowledge and engagement change among first-degree alters

Overall knowledge increased among first-degree alters (β = 0.008 (SE = 0.002), *p* = 0.001) (Table 1); specifically, knowledge of the *problem of,* and knowledge of *resources* for early childhood obesity increased significantly. Overall engagement did not significantly increase among first-degree alters. *Influence and power* significantly decreased during the study period and perceived *trust* significantly increased (β = 0.009 (SE 0.003), *p* = 0.004).

**Fig. 2:**
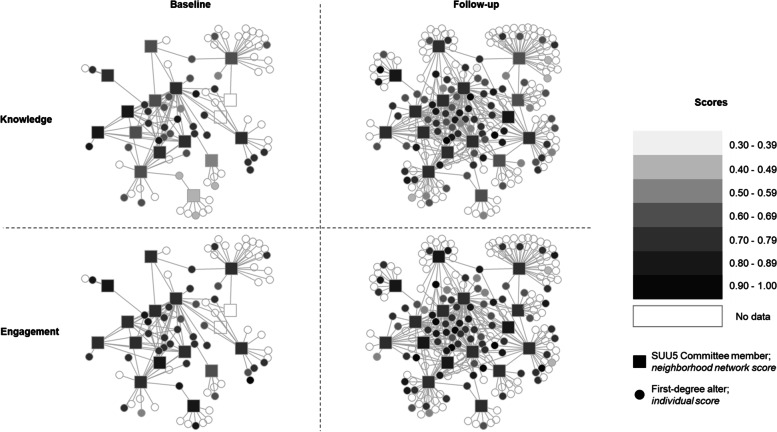
Network diagram showing levels of knowledge (top) and engagement (bottom) among network neighborhoods at baseline (left) and the end of the two-year pilot study (right)

Knowledge and engagement scores varied within neighborhood networks over time (Fig. 2 and Supplemental Table 1). We did not conduct hypothesis testing on changes within neighborhood networks due to the small size of several networks. The baseline network included 90 stakeholders and 131 ties, and the end of the study network included 217 stakeholders and 356 ties (Fig. 2).

### Hypothesis 3: Change in policies, systems, and environments

**Fig. 3:**
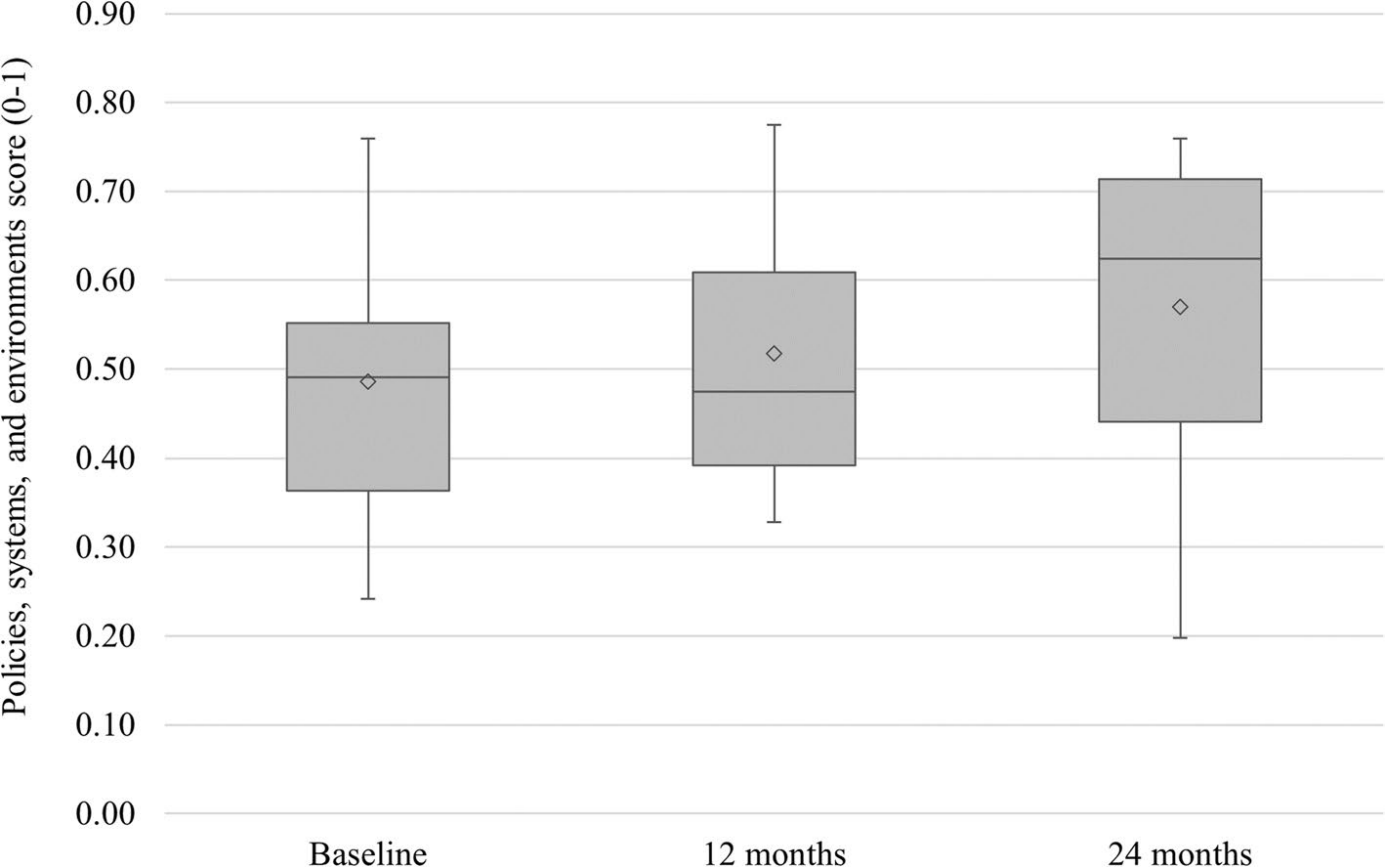
Box plot showing changes in Committee member (*N* = 16) reported policies, systems, and environments supportive of early childhood obesity prevention from baseline to the end of the two-year pilot study

Committee members reported improvements in policies, systems, and environments from baseline to 24 months (difference between means: 0.084; 95% CI: 0.028, 0.140; *p* = 0.003) (Fig. 3). The PSE survey type (A, A + B or A + B + C) did not affect whether Committee members perceived improvements in PSE. Changes in Committee members’ engagement scores were significantly associated with PSE score changes (correlation coefficient = 0.56, *p* = 0.037).

### Reach of an evidence-based community communication campaign

A total of 55 first-degree alters completed the campaign reach survey, 73% of whom reported seeing *Small Steps: Eat Play Sleep* campaign materials. The most frequently viewed campaign assets were the posters (83%), followed by brochures (75%), Mayor’s Wellness Challenge materials (38%), PowerPoint presentations (i.e., tip sheets for health and childcare providers) (13%) and other materials (8%). The most common setting to encounter campaign materials was the organization in which the respondent worked (58%), a school (38%), city of Somerville building (33%), library (20%), healthcare office (18%), playground (15%), WIC office (13%), childcare center (13%), farmers’ market (10%), and playgroup (10%).

## Discussion

We found that the SUU5 committee significantly increased knowledge of and engagement with early childhood obesity prevention and that knowledge of the topic increased within the members’ professional networks. With support from the research team, the committee designed, created, and disseminated materials for a community-wide communications campaign that provided consistent, evidence-based early childhood obesity prevention recommendations [35]. Committee members reported significant improvements in policies, systems, and environments in policies, systems, and environments that support early childhood obesity prevention at the conclusion of the two-year study. Changes in Committee members’ engagement were significantly associated with reported PSE improvements.

Our upstream intervention was designed to catalyze whole-of-community change by facilitating changes in knowledge, engagement, and relationships across sectors, and by using systems thinking methods to spark new “big picture” ideas for addressing childhood obesity and related disparities. A review of whole-of-community obesity prevention studies found that most studies reported improvements in health outcomes (e.g., reductions in BMI), behaviors (e.g., reduced sugar-sweetened beverage consumption, increased physical activity), and/or psychosocial outcomes (e.g., reduced depressive symptoms) [29], and another study found that whole-of-community obesity prevention approaches may be cost effective [48]. Importantly, a review of whole-of-community obesity prevention studies found that low socioeconomic position groups benefited as much or more compared to high socioeconomic position groups, suggesting that this approach is well-suited for addressing persistent weight-related disparities in developed countries [49]. The SUU5 Committee chose to create a communications campaign that was designed to promote consistent, evidence-based messages about early childhood obesity prevention to caregivers in the many settings where children spend their time. Social marketing is established as part of an ecological approach to childhood obesity prevention and can bolster the effects of other programs and policies operating in a community [50, 51]. The campaign developed by the Committee was an important demonstration that the SDCD-intervention could yield cross-sector collaboration towards evidence-based action that spanned multiple community settings.

Members of this research group used data presented in this report as input into an agent-based model that characterizes the interpersonal interactions that collectively comprise diffusion of knowledge and engagement over time [47]. They found that the model could reproduce patterns of knowledge and engagement diffusion presented in this report, providing confidence in the model’s explanatory power. Next, the researchers used the model for two types of extrapolation beyond the data presented here: they projected increases in both knowledge and engagement among the broader community (i.e., including those who were not study participants), and explored several counterfactual scenarios to gain insight into how different implementation strategies might have affected change in community knowledge and engagement. The study conducted by Kasman and colleagues offers an exciting example of how mathematical models can expand the scope of research questions that can be answered using data from a community-based intervention. Based in part on the data and analyses in this report, a future iteration of the agent-based model used by Kasman and colleagues could be extended beyond “upstream” changes in knowledge and engagement to explore the causal mechanisms that might translate these to changes in PSE.

### Strengths

Engagement was fostered by using CBSD, GMB, and GMB-like activities during Committee meetings, and a strength of this approach includes facilitation techniques that promote group participation and power leveling (e.g., requesting each participant speaks at least once during activities, participants sharing one idea at a time, preventing dominant voices overtaking conversation) [32]. Engagement was highest among Committee members after meetings where they worked on the campaign design and dissemination plans showing the power of engaging leaders in obesity prevention design [35]. As engagement increased over the course of the intervention, increases in PSE accelerated as well. This finding may reflect Committee members’ perceptions that their communication campaign improved PSEs in Somerville, and/or Committee members’ engagement with the topic of early childhood obesity may have translated into additional improvements in PSEs that support early childhood obesity prevention.

### Limitations

The study design did not include a comparison group so we cannot rule out whether other factors beyond the SDCD theory-informed intervention influenced observed changes in knowledge and engagement. Future studies should include a comparison group (e.g., quasi-experimental design with a wait-list comparison group) to isolate the effects of the intervention on participants. The Committee and first degree alters were predominantly highly educated, white, and female, reflecting demographic patterns in the broader fields of public health and nutrition [52, 53]. Efforts to diversify the fields [54] and the stakeholder groups this research group partners with are underway. Missing data from first-degree alters made it difficult to fully capture potential diffusion of knowledge and engagement throughout the community. We addressed this limitation by carrying first-degree alters forward to subsequent waves of data collection and by imputation, allowing alters to remain part of the network from their first nomination. If last observation carried forward was not possible (e.g., if respondent was missing initial survey) mean imputation was used replacing the missing value with the mean of remaining values. Our study underscores a need to for new, creative methods for collecting social network data that provides a more complete network sample. Researchers are experimenting with apps, administrative data, and records to collect network data without asking participants to complete lengthy surveys [55]. While the PSE survey assessed Committee members’ perceptions, more objective measures of PSE change would strengthen future studies. Existing data capturing PSE and child health outcomes could be utilized to assess the effects of SDCD theory-informed interventions and similar coalition-led initiatives.

### Implications

These findings highlight a potential mechanism through which community coalitions and similar groups affect positive change related to children’s health in their communities.

## Conclusion

SUU5’s SDCD-informed intervention increased knowledge of, and engagement with, early childhood obesity prevention among a multi-sector group of stakeholders, and improvements in those constructs diffused into some professional network clusters. Increases in engagement among the group were also associated with improvements in perception of policies, systems, and engagement related to early childhood obesity prevention in the community. The SDCD theory [21, 22], data collection instruments [20, 38], effects on community-based networks [36, 56], agent-based modeling [47], and interventions advances the science of effective obesity prevention research in communities [35, 57].

## Supplementary Information

**Additional file 1:** **Supplemental Table 1.** Knowledge scores among Shape Up Under 5 neighborhood networks at each data collection point over the two-year pilot study. Mean (SD) knowledge and engagement scores range from 0 (low) to 1 (high). Neighborhood networks sizes (*n*) increase cumulatively over time. **Supplemental Table 2.** Engagement scores among Shape Up Under 5 neighborhood networks at each data collection point over the two-year pilot study. Mean (SD) knowledge and engagement scores range from 0 (low) to 1 (high). Neighborhood networks sizes (*n*) increase cumulatively over time.

## Data Availability

The datasets used and/or analyzed during the current study are available from the corresponding author on reasonable request.
