# Supplementary material for: Community coalition efforts to prevent childhood obesity: two-year results of the Shape Up Under 5 study
Source: BMC Public Health. 2023 Mar 20;23:529. doi: 10.1186/s12889-023-15288-5 (PMC10026415; doi:10.1186/s12889-023-15288-5)
Supplement: Supplementary file 1 — Additional file 1: Supplemental Table 1. Knowledge scores among Shape Up Under 5 neighborhood networks at each data collection point over the two-year pilot study. Mean (SD) knowledge and engagement scores range from 0 (low) to 1 (high). Neighborhood networks sizes (n) increase cumulatively over time. Supplemental Table 2. Engagement scores among Shape Up Under 5 neighborhood networks at each data collection point over the two-year pilot study. Mean (SD) knowledge and engagement scores range from 0 (low) to 1 (high). Neighborhood networks sizes (n) increase cumulatively over time. [file 12889_2023_15288_MOESM1_ESM.docx]

Supplemental Table 1. Knowledge scores among Shape Up Under 5 neighborhood networks at each data collection point over the two-year pilot study. Mean (SD) knowledge and engagement scores range from 0 (low) to 1 (high). Neighborhood networks sizes (*n*) increase cumulatively over time.

| **Neighborhood knowledge scores** | | | | | | | | | | | |
| --- | --- | --- | --- | --- | --- | --- | --- | --- | --- | --- | --- |
| **ID** | **Baseline** | ***n*** | **6 months** | ***n*** | **12 months** | ***n*** | **18 months** | ***n*** | **24 months** | ***n*** |  |
| 1 | 0.67 (0.06) | 3 | 0.71 (0.07) | 6 | 0.74 (0.06) | 8 | 0.78 (0.09) | 9 | 0.79 (0.07) | 9 |  |
| 2 | 0.71 (0.08) | 14 | 0.73 (0.09) | 21 | 0.72 (0.08) | 28 | 0.73 (0.11) | 34 | 0.74 (0.12) | 35 |  |
| 3 | 0.73 (0.00) | 2 | 0.73 (0.05) | 4 | 0.80 (0.13) | 4 | 0.73 (0.04) | 4 | 0.83 (0.12) | 4 |  |
| 4 | 0.75 (0.01) | 7 | 0.75 (0.09) | 8 | 0.77 (0.10) | 10 | 0.76 (0.09) | 12 | 0.76 (0.09) | 14 |  |
| 5 | 0.73 (0.10) | 4 | 0.72 (0.09) | 17 | 0.74 (0.09) | 22 | 0.74 (0.12) | 23 | 0.75 (0.11) | 23 |  |
| 6 | 0.50 (0.02) | 2 | 0.53 (0.06) | 2 | 0.58 (0.08) | 2 | 0.66 (0.09) | 2 | 0.65 (0.13) | 3 |  |
| 7 | - ^a^ | 0 | 0.71 (0.17) | 4 | 0.66 (0.13) | 4 | 0.64 (0.09) | 4 | 0.64 (0.12) | 5 |  |
| 8 | 0.64 (0.04) | 4 | 0.69 (0.06) | 4 | 0.68 (0.09) | 4 | 0.62 (0.11) | 8 | 0.61 (0.15) | 8 |  |
| 9 | - ^a^ | 0 | 0.78 (0.02) | 3 | 0.75 (0.06) | 7 | 0.81 (0.07) | 8 | 0.81 (0.07) | 9 |  |
| 10 | 0.73 (0.00) | 2 | 0.79 (0.07) | 4 | 0.74 (0.08) | 6 | 0.79 (0.09) | 7 | 0.80 (0.09) | 7 |  |
| 11 | 0.67 (0.09) | 7 | 0.71 (0.10) | 13 | 0.69 (0.08) | 19 | 0.73 (0.10) | 21 | 0.74 (0.10) | 21 |  |
| 12 | 0.69 | 1 | 0.81 (0.07) | 4 | 0.79 (0.05) | 5 | 0.76 (0.06) | 6 | 0.79 (0.06) | 7 |  |
| 13 | 0.43 (0.00) | 2 | 0.54 (0.00) | 3 | 0.63 (0.13) | 3 | 0.66 (0.10) | 3 | 0.66 (0.10) | 3 |  |
| 14 | 0.68 (0.11) | 9 | 0.68 (0.10) | 16 | 0.69 (0.13) | 16 | 0.71 (0.12) | 17 | 0.72 (0.13) | 17 |  |
| 15 | 0.80 (0.00) | 3 | 0.74 (0.09) | 10 | 0.73 (0.05) | 12 | 0.74 (0.09) | 13 | 0.76 (0.09) | 13 |  |
| 16 | 0.80 (0.00) | 4 | 0.70 (0.07) | 14 | 0.70 (0.07) | 15 | 0.71 (0.08) | 15 | 0.72 (0.09) | 15 |  |
| ^a^ Committee member did not report any ties at this round of data collection | | | | | | | | | | |  |

Supplemental Table 2. Engagement scores among Shape Up Under 5 neighborhood networks at each data collection point over the two-year pilot study. Mean (SD) knowledge and engagement scores range from 0 (low) to 1 (high). Neighborhood networks sizes (*n*) increase cumulatively over time.

| **Neighborhood engagement scores** | | | | | | | | | | | |
| --- | --- | --- | --- | --- | --- | --- | --- | --- | --- | --- | --- |
| **ID** | **Baseline** | ***n*** | **6 months** | ***n*** | **12 months** | ***n*** | **18 months** | ***n*** | **24 months** | ***n*** |  |
| 1 | 0.80 (0.06) | 3 | 0.83 (0.09) | 6 | 0.79 (0.07) | 8 | 0.83 (0.09) | 9 | 0.82 (0.09) | 9 |  |
| 2 | 0.74 (0.06) | 14 | 0.80 (0.09) | 21 | 0.76 (0.09) | 28 | 0.77 (0.10) | 34 | 0.76 (0.10) | 35 |  |
| 3 | 0.86 (0.00) | 2 | 0.84 (0.05) | 4 | 0.76 (0.01) | 4 | 0.74 (0.05) | 4 | 0.82 (0.04) | 4 |  |
| 4 | 0.76 (0.10) | 7 | 0.77 (0.20) | 8 | 0.81 (0.16) | 10 | 0.77 (0.14) | 12 | 0.77 (0.12) | 14 |  |
| 5 | 0.76 (0.04) | 4 | 0.80 (0.08) | 17 | 0.78 (0.09) | 22 | 0.80 (0.09) | 23 | 0.80 (0.09) | 23 |  |
| 6 | 0.64 (0.02) | 2 | 0.63 (0.11) | 2 | 0.64 (0.03) | 2 | 0.67 (0.12) | 2 | 0.76 (0.05) | 3 |  |
| 7 | - ^a^ | 0 | 0.76 (0.14) | 4 | 0.80 (0.10) | 4 | 0.81 (0.07) | 4 | 0.72 (0.10) | 5 |  |
| 8 | 0.74 (0.05) | 4 | 0.74 (0.10) | 4 | 0.74 (0.09) | 4 | 0.70 (0.12) | 8 | 0.72 (0.12) | 8 |  |
| 9 | - ^a^ | 0 | 0.76 (0.10) | 3 | 0.75 (0.06) | 7 | 0.81 (0.07) | 8 | 0.80 (0.08) | 9 |  |
| 10 | 0.86 (0.00) | 2 | 0.89 (0.03) | 4 | 0.84 (0.08) | 6 | 0.87 (0.09) | 7 | 0.88 (0.08) | 7 |  |
| 11 | 0.74 (0.07) | 7 | 0.78 (0.08) | 13 | 0.75 (0.09) | 19 | 0.78 (0.11) | 21 | 0.79 (0.10) | 21 |  |
| 12 | 0.76 | 1 | 0.87 (0.09) | 4 | 0.84 (0.09) | 5 | 0.81 (0.08) | 6 | 0.84 (0.07) | 7 |  |
| 13 | 0.86 (0.00) | 2 | 0.79 (0.00) | 3 | 0.77 (0.10) | 3 | 0.77 (0.10) | 3 | 0.77 (0.10) | 3 |  |
| 14 | 0.74 (0.07) | 9 | 0.80 (0.07) | 16 | 0.76 (0.09) | 16 | 0.77 (0.10) | 17 | 0.76 (0.03) | 17 |  |
| 15 | 0.76 (0.03) | 3 | 0.81 (0.09) | 10 | 0.77 (0.11) | 12 | 0.78 (0.12) | 13 | 0.78 (0.12) | 13 |  |
| 16 | 0.76 (0.03) | 4 | 0.78 (0.07) | 14 | 0.71 (0.09) | 15 | 0.74 (0.09) | 15 | 0.74 (0.09) | 15 |  |
| ^a^ Committee member did not report any ties at this round of data collection | | | | | | | | | | |  |
